# Mining the bitter melon (*momordica charantia *l.) seed transcriptome by 454 analysis of non-normalized and normalized cDNA populations for conjugated fatty acid metabolism-related genes

**DOI:** 10.1186/1471-2229-10-250

**Published:** 2010-11-16

**Authors:** Peizhen Yang, Xiangjun Li, Matthew J Shipp, Jay M Shockey, Edgar B Cahoon

**Affiliations:** 1 Donald Danforth Plant Science Center, 975 North Warson Road, Saint Louis, Missouri 63132 USA; 2 Department of Biochemistry and Center for Plant Science Innovation, George W. Beadle Center, 1901 Vine Street, University of Nebraska-Lincoln, Lincoln, Nebraska 68588 USA; 3 Commodity Utilization Research Unit, USDA-ARS, Southern Regional Research Center, New Orleans, LA 70124 USA

## Abstract

**Background:**

Seeds of *Momordica charantia *(bitter melon) produce high levels of eleostearic acid, an unusual conjugated fatty acid with industrial value. Deep sequencing of non-normalized and normalized cDNAs from developing bitter melon seeds was conducted to uncover key genes required for biotechnological transfer of conjugated fatty acid production to existing oilseed crops. It is expected that these studies will also provide basic information regarding the metabolism of other high-value novel fatty acids.

**Results:**

Deep sequencing using 454 technology with non-normalized and normalized cDNA libraries prepared from bitter melon seeds at 18 DAP resulted in the identification of transcripts for the vast majority of known genes involved in fatty acid and triacylglycerol biosynthesis. The non-normalized library provided a transcriptome profile of the early stage in seed development that highlighted the abundance of transcripts for genes encoding seed storage proteins as well as for a number of genes for lipid metabolism-associated polypeptides, including Δ12 oleic acid desaturases and fatty acid conjugases, class 3 lipases, acyl-carrier protein, and acyl-CoA binding protein. Normalization of cDNA by use of a duplex-specific nuclease method not only increased the overall discovery of genes from developing bitter melon seeds, but also resulted in the identification of 345 contigs with homology to 189 known lipid genes in Arabidopsis. These included candidate genes for eleostearic acid metabolism such as diacylglycerol acyltransferase 1 and 2, and a phospholipid:diacylglycerol acyltransferase 1-related enzyme. Transcripts were also identified for a novel *FAD2 *gene encoding a functional Δ12 oleic acid desaturase with potential implications for eleostearic acid biosynthesis.

**Conclusions:**

454 deep sequencing, particularly with normalized cDNA populations, was an effective method for mining of genes associated with eleostearic acid metabolism in developing bitter melon seeds. The transcriptomic data presented provide a resource for the study of novel fatty acid metabolism and for the biotechnological production of conjugated fatty acids and possibly other novel fatty acids in established oilseed crops.

## Background

A target of plant biotechnology has been the engineering of novel fatty acid production in seeds of established crops to enhance the industrial value of vegetable oils [1]. This research has involved the identification of genes for the synthesis of novel fatty acids from non-agronomic species and the subsequent transfer of these genes to crops for seed-specific expression. Targets for this research have included epoxy and hydroxylated fatty acids [1-3]. With only a few exceptions, these efforts have resulted in the production of novel fatty acids at levels significantly lower than those found in native sources. The modest success of this research has underscored the lack of knowledge in the specialized metabolism associated with the production and storage of novel fatty acids in oilseeds.

Our research has centered on fatty acids containing conjugated, or non-methylene interrupted double bonds, as a system for addressing gaps in our understanding of novel fatty acid metabolism. Oils enriched in conjugated fatty acids can be used as drying agents in coating materials such as paints, inks, and varnishes. The conjugated double bonds of these fatty acids are highly prone to oxidation, which enhances rates of polymerization or "drying" of coating materials [4]. The most widely used oil for these applications is tung oil extracted from seeds of *Vernicia fordii*. The value of this oil as a drying agent arises from its high content of the conjugated fatty acid α-eleostearic acid (18:3 Δ^9*cis*, 11*trans*, 13*trans*^) that comprises > 80% of tung oil [5]. Eleostearic acid also comprises ~65% of the seed oil of *Momordica charantia *(bitter melon) [6]. Other conjugated fatty acids, including calendic (18:3 Δ^8*trans*,10*trans*,12*cis*^), catalpic (18:3 Δ^9*trans*,11*trans*,13*cis*^), and punicic (18:3 Δ ^9*cis*,11*trans*,13*cis*^) acids, can be found in seed oils from species of at least nine different plant families [5,7-9].

Efforts to transfer eleostearic acid production to seeds of temperate crops have been facilitated by the identification of genes encoding variant forms of the Δ12 oleic acid desaturase (or FAD2) termed "conjugases" [10-12]. These enzymes catalyze the removal of hydrogen atoms from the carbon atoms that flank the Δ12 double bond of linoleic acid, and convert the Δ12 double bond into two conjugated Δ11, Δ13 double bonds [10]. The product of this reaction is a conjugated triene with Δ9, 11, 13 unsaturation. In addition to Δ12-specific conjugases, Δ9 conjugases have been described in *Calendula officinalis *and *Dimorphotheca sinuata *that convert the Δ9 double bond of linoleic acid into conjugated Δ8, Δ10 double bonds [13-15].

Transgenic expression of Δ9 and Δ12 conjugase genes under control of strong seed-specific promoters in Arabidopsis and soybean have yielded conjugated fatty acid levels of 10 to 15% of the total seed oils [9]. These levels are well below amounts of conjugated fatty acids that naturally accumulate in seeds of plants such as tung and bitter melon. In the engineered Arabidopsis and soybean seeds, conjugated fatty acids not only accumulate in storage form in triacylglycerols (TAGs) but are also detected in aberrantly high amounts in membrane phospholipids (10% to 25% of the total fatty acids of these lipids), especially phosphatidylcholine [9]. In contrast, conjugated fatty acids are only minor components of phospholipids (< 1.5% of the total phospholipid fatty acids) in seeds from plants that naturally accumulate conjugated fatty acids to levels approaching 85% of the total fatty acids [9,16].

Although conjugases are of central importance for producing conjugated fatty acids, these results indicate that additional enzymes are required for the metabolism and accumulation of conjugated fatty acids in seeds of transgenic plants. Similar conclusions have been reached in efforts to engineer the production of hydroxy, epoxy, and acetylenic fatty acids in seeds [1,17-19]. These fatty acids are also produced by variant forms of the Δ12 oleic acid desaturase. As with conjugases, the variant FAD2 hydroxylases, epoxygenases, and acetylenases use fatty acids bound to phosphatidylcholine and possibly other phospholipids, such as phosphatidylethanolamine, as substrates [16,20,21]. The products of these enzymes must be efficiently metabolized from phospholipids for storage at high levels in TAGs. This can occur either by the direct removal of the unusual fatty acid from phosphatidylcholine or by removal of the phosphocholine head group of phosphatidylcholine to produce the diacylglycerol for TAG synthesis [1]. Findings from seeds engineered with conjugases and well as with acetylenases suggest that specialized enzymes have evolved for the metabolism of unusual fatty acids from their site of synthesis on phosphatidylcholine to their storage in TAGs [9,19]. These enzymes are presumably absent from seeds of plants such as Arabidopsis and soybean that do not normally produce unusual fatty acids. These may include specialized phospholipases, acyltransferases, and enzymes associated with the removal or transfer of phospholipid head groups.

The production of high levels of conjugated fatty acids and other unusual fatty acids formed by FAD2 variants in seeds of transgenic plants will undoubtedly require the identification of genes for these specialized metabolic enzymes. To facilitate this effort, we have undertaken 454 pyrosequencing studies to obtain a comprehensive profile of the transcriptome of developing bitter melon seeds during a period of rapid synthesis and accumulation of eleostearic acid. Bitter melon seeds offer a useful system to study the functional genomics of eleostearic acid synthesis relative to tung seeds, which accumulate higher levels of this fatty acid, because bitter melon plants can be grown under controlled conditions and seeds can be more easily staged for eleostearic acid accumulation. As described here, we have identified ~14,000 unique gene transcripts from normalized and non-normalized cDNA populations, including transcripts for the majority of enzymes involved in lipid biosynthesis and metabolism. Candidate genes for potential enzymes involved in eleostearic acid metabolism are highlighted, and also a divergent class of FAD2 that may be specialized for eleostearic acid biosynthesis in bitter melon seeds is described.

## Results and Discussion

### Determination of a seed developmental stage for rapid biosynthesis of eleostearic acid

**Figure 1:**
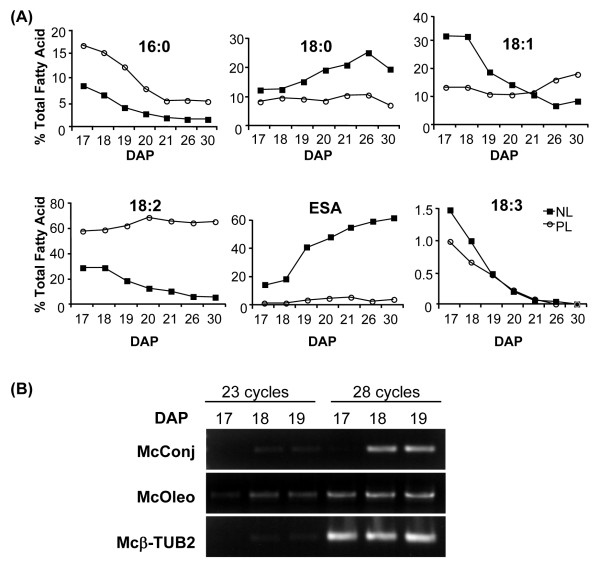
**Fatty acid compositions of lipids and detection of selected transcripts at different stages of bitter melon seed development**. (A). Distribution of fatty acids in phospholipids (PL, *open circle*) and the triacylglycerol (TAG)-enriched neutral lipids (NL, *black square*) during the development of bitter melon. The fatty acids shown are palmitic acid, 16:0; stearic acid, 18:0; oleic acid, 18:1; linoleic acid, 18:2; linolenic acid, 18:3; and eleostearic acid, ESA. (B). RT-PCR detection of transcripts for the bitter melon conjugase (McConj) and oleosin (McOleo) during bitter melon seed development. β-Tubulin (Mcβ-Tub2)-specific primers were used as a control to assess the quality of first-strand cDNA. The seeds were collected at the designated days after pollination (DAP).

Bitter melon seeds were initially analyzed at different time points after floral pollination to determine the developmental stages at which active synthesis and accumulation of eleostearic acid occur. It was anticipated that this information would provide the basis for selection of an optimal time point during seed development for the identification of genes associated with eleostearic acid metabolism. For these studies, lipids were extracted from developing seeds, consisting primarily of embryo and progressively lesser amounts of endosperm during seed development. Developing seeds were sampled at intervals from 17 to 30 days after pollination (DAP). The extracted lipids were partitioned by silica solid phase extraction into fractions of neutral lipids, comprised primarily of triacylglycerols (TAGs), and phospholipids. From analysis of fatty acids from these fractions, it was observed that rapid accumulation of eleostearic acid begins between 18 and 19 DAP, and the accumulation of eleostearic acid is detected almost exclusively in the TAG-enriched neutral lipid fraction. This accumulation coincides with the growth and expansion of the embryo. Accompanying the accumulation of eleostearic acid are large decreases in palmitic acid (16:0) and α-linolenic acid (18:3) content in neutral and phospholipids and large increases in stearic acid (18:0) content in the neutral lipids. Consistent with the transition in fatty acid profile, expression of genes for the bitter melon conjugase, which produces eleosteraric acid, and the oil body-associated oleosin was detected by RT-PCR initially at 18 DAP (Figure 1B). These collective data suggest that seeds at 18 DAP are enriched in the transcripts for enzymes involved in the synthesis and accumulation of eleostearic acid. This stage in seed development therefore is a suitable time point for transcriptomic analyses to identify metabolic genes specialized for eleostearic acid accumulation.

### Construction of non-normalized and normalized cDNA libraries

Transcriptomic studies were conducted to identify genes associated with the synthesis and metabolism of eleostearic acid in bitter melon seeds. From previous expressed sequence tag analysis of developing bitter melon seeds [10], it was known that genes for enzymes such as acyltransferases that may be specialized for eleostearic acid metabolism are not highly expressed in developing seeds of this plant. Therefore, in order to enhance gene discovery, 454 pyrosequencing was conducted using non-normalized and normalized cDNA populations prepared from bitter melon seeds at 18 DAP. 454 pyrosequencing is now an established platform for deep sequencing of genomes and transcriptomes, and normalization of cDNAs was anticipated to enrich for low abundance mRNA transcripts in developing bitter melon seeds.

**Figure 2:**
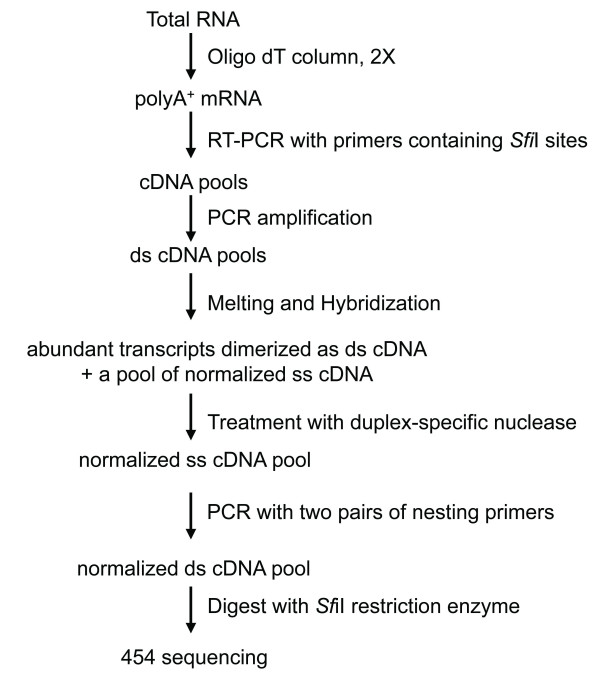
**Preparation of normalized cDNA library from total RNA**. Abbreviations: ss cDNA, single-stranded cDNA; ds cDNA, double-stranded cDNA.

Prior efforts to normalize cDNA libraries have involved a number of different protocols including those based on the use of subtractive hybridization, hydroxyapatite (HAP)-bound column chromatography, and duplex-specific nuclease (DSN) treatment [22-24]. The normalization procedure used (as outlined in Figure 2) was adapted from the Evrogen Trimmer-Direct kit that uses kamchatka crab-based DSN to reduce amounts of abundant cDNAs that more rapidly re-anneal following melting and hybridization. This kit is also compatible with the Clontech SMART cDNA library, allowing for cloning of normalized cDNA inserts into a bacterial vector. This method has been previously used to normalize cDNA libraries from a number of organisms including lake sturgeon, asian seabass, and Medicago [25-27].

**Figure 3:**
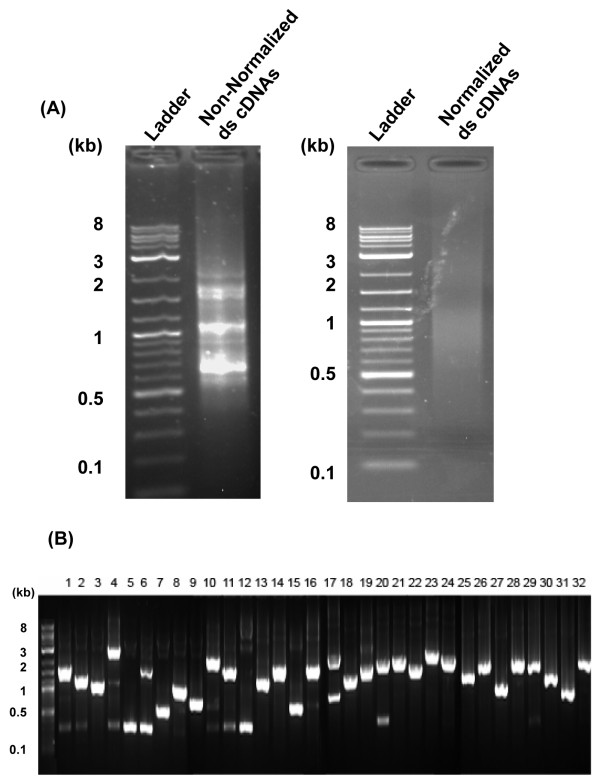
**Illustration of normalized versus non-normalized library on agarose gel electrophoresis**. (A). Analysis of equal loadings of non-normalized and normalized cDNA populations on a 1% agarose gel. (B). DNA insert fragments were amplified by PCR from random individual colonies in the normalized cDNA library, and analyzed on a 1% agarose gel. The 1 kb DNA ladder was loaded as control.

Prior to the 454 sequencing, a pilot experiment was conducted to analyze the composition of transcripts from both non-normalized and normalized cDNA pools by sequencing of clones from each library. From the non-normalized cDNA library, 40 independent colonies were isolated and sequenced (Additional File 1). About 40% of these sequences were found to encode seed storage proteins such as napin- and trypsin inhibitor-related polypeptides. In the normalized cDNA library, a large reduction in percentage of the abundant transcripts encoding seed storage proteins and a wider range in size of transcripts were observed (Figure 3). The sequencing results from the sequences derived from the normalized library revealed many transcripts unrelated to the seed storage proteins, confirming the effectiveness of normalization (Additional File 1).

**Figure 4:**
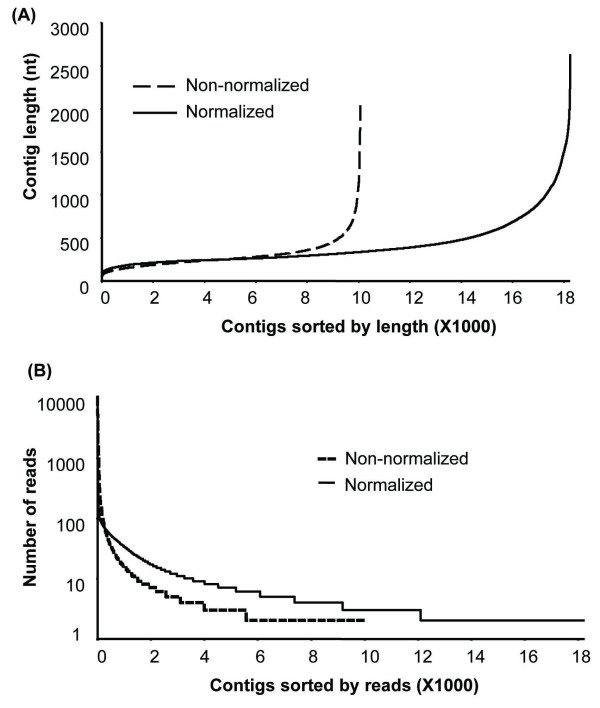
**Distribution of sequence length (A) and number of reads (> = 2) (B) of contigs in both non-normalized and normalized cDNA populations**.

Following the pilot study, transcript sequences were analyzed in both the normalized and non-normalized cDNA populations using 454 deep sequencing. The initial run of 454 pyrosequencing generated 404,468 reads from the non-normalized cDNA pools and 255,687 reads from the normalized cDNA pools (Table 1). After trimming and screening, about 228,000 and 177,991 clean reads remained in the non-normalized and normalized cDNA pools, respectively. About 22% singletons were found in both populations. The remaining 78% reads were assembled into 10,072 and 18,245 contigs from non-normalized and normalized cDNA population, respectively (Figure 4A and 4B). In both libraries, the average length of ESTs was approximately 800 nucleotides (Table 1). The extra 80% assembled contigs in the normalized cDNA pools suggests that normalization played a critical role in enriching low-abundant unique transcripts and increasing the total number of cDNAs.

**Table 1:** Assembly statistics of non-normalized and normalized cDNA libraries.

	Non-normalized	Normalized
Number of sequences	404,468	255,687
Number of high quality sequences	281,933	216,950
Number of the clean reads	228,000	177,991
Number of singlets	53,897	38,959
	(23.6%)	(21.8%)
Number of contigs	10,072	18,245
Average length of contigs	297	414
Average length of EST	828	796
Average read of contigs	23	10
Number of contigs match Viridiplants	4,459	11,558
Number of contigs match Arabidopsis	3,643	11,211
(# of unique homologs in Arabidopsis)	(2,187)	(6,737)
Lipid genes contigs	193	345
(# of unique homologs for known Arabidopsis lipid genes)	(85)	(189)

These assembled contigs were then searched against both Arabidopsis TAIR7 and viridiplantae subdivision of NCBI protein database with e-value cutoff of 1e-10, to find their homologues using BLASTX program. In both libraries, about 50% of the contigs assembled did not have a match in either database (designated "no hit" transcripts). In the normalized cDNA library, about half of these "no hit" sequences were fragments between 200 and 300 nucleotides (nt). For transcript contigs smaller than 200 nt, about 74% were "no hit". In contrast, 90% of transcripts at 500-1,000 nt and 99% of transcripts longer than 1,000 nt, were identified with homologs in Arabidopsis and/or green plant genomes. The majority of these "no hit" sequences probably result from primer dimer and holopolymer formation casuing the short sequences not to give high matches to known proteins. These could also encode 5'- and 3'- untranslated regions of transcripts, or small RNA species that do not encode proteins. The number of transcripts generated from the normalized library with either Viridiplant or Arabidopsis homologues was almost three times more than from the non-normalized populations (Table 1). This, as well as the observation that normalization yielding 80% more contigs, strongly suggests that normalization has played an important role in increasing the detection of low-abundance transcripts and that the increase in the total number of unique transcripts will facilitate the gene mining processes. These sequences are publicly available for web-based BLAST searches at http://genomics.msu.edu/JO/blast/blast.html.

### Non-normalized cDNA populations reflect an early stage of seed development

Deep sequencing of the non-normalized cDNAs allowed for analysis of gene expression profiles during an early stage of seed development in bitter melon. After comparing each contig with the non-redundant (nr) protein database of the NCBI and Arabidopsis proteins at TAIR using the BLASTX program, 4,459 contigs (representing 152,989 total reads) were identified with 3,093 unique Viridiplant homologs. Among the most abundant 50 contigs in the library, 20 of them had no identified homologs (Additional File 2). The remaining contigs encoded primarily seed storage protein- or ribosome-inactivating protein-related polypeptides.

To further focus the analysis of developing seeds of bitter melon, contigs with homologs for the same gene were combined as a single cluster and used to rank the most abundant sequences (> 250 read counts) in the non-normalized cDNA population. The most abundant sequences encoded seed storage proteins, which represented 25% of the total contigs (Table 2; Additional File 2). These included transcripts for genes related to napin (most abundant, 169 contigs with 23,678 reads), napin-like protein large chain, legumin-like seed storage protein, and 11 S globulin and its precursor. The second group of highly expressed genes (about 15% of total) encode ribosome-inactivating proteins, including type I and type II, MAP30, α-momorcharin [28], trichosanthin and its precursor, and NeuAc-gal/GalNAc-binding lectin.

**Table 2:** List of gene products for the 50 most abundant transcript reads in the non-normalized cDNA library from bitter melon seeds.

Homologs	# of reads	# of contigs	Gene products
gi|21327881	23,678	169	Napin
gi|29243138	15,142	34	hypothetical protein
gi|11225518	8,197	45	MAP30
gi|56788031	7,564	15	seed storage protein, legumin-related
gi|19528	6,861	60	α-momorcharin
gi|167492	6,340	42	11 S globulin beta-subunit precursor
gi|13171073	6,309	3	ribosome-inactivating protein precursor
gi|147798373	5,370	10	hypothetical protein BURP domain
gi|3808062	4,278	42	cupin 2, PV100
gi|976231	2,438	3	ribonuclease (RNase LC1)
gi|157343662	1,921	6	unnamed protein product, PEBP
gi|29165641	1,804	5	trichosanthin precursor
gi|109895116	1,733	5	ribosome-inactivating protein
gi|15215752	1,473	5	At5g59750, riboflavin biosynthesis protein
gi|5381325	1,346	8	11 S globulin precursor
gi|4106063	1,282	8	trichoanguin
gi|157358487	1,121	6	unnamed protein product
gi|28371823	1,012	15	Δ12 oleate desaturase, FAD2/FAD2v
gi|157335527	1,000	3	class-3 lipase
gi|118482397	833	3	dehydroascorbate reductase 2
gi|170444	780	1	extensin (class II)
gi|1838961	714	2	acyl carrier protein
gi|118482623	637	2	γ-thionin family protein
gi|157345129	616	1	class-3 lipase
gi|1938236	571	1	acyl-CoA-binding protein
gi|164449275	565	6	caleosin
gi|157327440	533	1	cytochrome P450
gi|1944181	492	4	aspartic endopeptidase
gi|29243128	479	6	putative major latex protein
gi|157340924	418	1	oxygenase
gi|1911803	407	36	napin-like protein large chain
gi|1666094	402	4	gibberellin 20-oxidase
gi|691752	379	5	cupin 2 preproMP27-MP32
gi|156891145	374	2	glutaredoxin
gi|157338378	363	1	Sec 61 protein
gi|32170831	357	2	leaf ubiquitous urease
gi|49532940	357	3	type-2 metallothionein
gi|10129818	332	15	ribosome inactivating protein type I
gi|19338630	311	3	48-kDa glycoprotein precursor
gi|157329758	293	4	DUF588 unnamed protein product
gi|433609	292	1	enolase
gi|224797	291	2	urease
gi|6224716	284	3	Δ12 oleic acid desaturase-like, conjugase
gi|2331046	281	4	type 2 ribosome-inactivating protein precursor
gi|1587207	271	12	NeuAc-gal/GalNAc-binding lectin
gi|87162930	269	2	lipoxygenase, embryo-specific 3
gi|1669585	269	3	cytosolic ascorbate peroxidase
gi|118484529	266	4	At2g37600, Ribosomal L36e
gi|2224892	260	1	gibberellin 7-oxidase
gi|70724314	258	4	cytochrome P450 monooxygenase

The numbers of reads and contigs and most related homolog for each gene product are also shown.

Also abundant in non-normalized cDNAs from bitter melon seeds at 18 DAP were reads for transcripts encoding structural proteins associated with cell development, including latex protein, extensin, ribosomal associated membrane protein 4 (RAMP4), glycoprotein, and sec61 protein (Table 2; Additional File 2). Genes involved in gibberellin biosynthesis, such as gibberellin 20-oxidase and gibberellin 7-oxidase, are highly expressed in the early developmental stage of bitter melon seeds. Homologs for these genes were previously shown to be upregulated during the fruit maturation of morning glory [29]. Highly expressed genes were also detected for polypeptides involved in redox balance in seeds, including riboflavin biosynthase, oxygenase, cytochrome P450, glutaredoxin, type-2 metallothionein, cytosolic ascorbate peroxidase, and cytochrome P450 monoxygenase.

### Transcripts for many lipid-related genes are abundant in developing bitter melon seeds

Genes encoding enzymes and other polypeptides associated with lipid biosynthesis and metabolism were detected among the 50 most abundant reads from the non-normalized cDNAs (Table 2; Additional File 2). These included lipoxygenase, Δ12 oleic acid desaturase (FAD2), Δ12 fatty acid conjugase, and Class-3-type lipases. The most abundant reads included those encoding lipid or fatty acid-binding proteins, such as a phosphatidylethanolamine binding protein (PEBP)-homolog, acyl-CoA-binding protein, and acyl-carrier protein. Interestingly, sequences for PEBP (gi|157343662) (1,921 reads and 6 contigs) were the 11^th ^most abundant in the non-normalized cDNAs, PEBP has been implicated in signal transduction in mammalian systems, and its role in eleostearic acid metabolism, if any, is not clear.

Genes for oil body-associated proteins were also highly represented in the bitter melon seed transcriptome. Most notably, 212 reads representing three contigs were detected for caleosin genes. Of lesser abundance were oleosin genes representing homologs of Arabidopsis *OLEO1 *and *OLEO4 *(81 reads in two contigs, and 16 reads in one contig were detected for *OLEO1 *and *OLEO4 *homologs, respectively). Given the variability of the amphipathic domain found in oleosins from diverse sources [30,31], it is possible that one or more of the bitter melon oleosins has specificity for eleostearic-rich TAGs to promote their efficient packaging and accumulation in oil bodies.

Reads for Class 3 TAG lipase were also detected at high abundance in the non-normalized cDNA pool (Table 2). Two groups of these homologs were present in the bitter melon libraries: (gi|157335527 with 1,000 reads, 3 contigs) and (gi|157345129 with 616 reads, 1 contig). Although the role for Class 3 TAG lipases in developing bitter melon seeds is not known, transcripts for this enzyme class were also found to be abundant in developing castor bean seeds (*Ricinus communis*) endosperm [32].

### Normalization enhances gene discovery in developing bitter melon seeds

A major goal of this study was to deeply mine the transcriptome of developing bitter melon seeds for fatty acid biosynthetic and metabolic genes. From this pool, candidate genes that are specialized for eleostearic metabolism can be identified. Normalization of the bitter melon cDNA pools was employed as a technique to facilitate gene discovery efforts.

**Figure 5:**
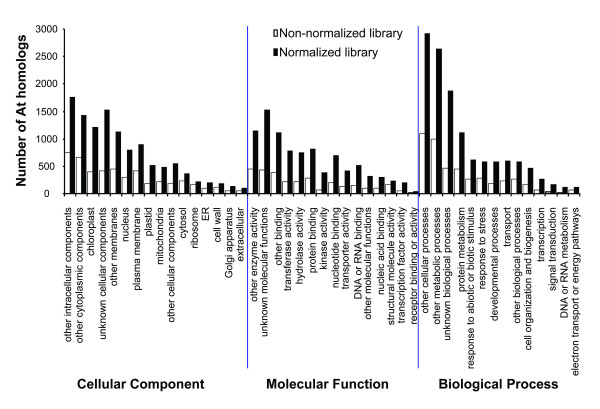
**Go function analysis **[55]**of Arabidopsis gene homologous from both non-normalized and normalized cDNA libraries**. Distribution of genes associated with different cellular components, molecular functions and biological processes.

To assess the efficacy of normalization, the bitter melon sequences from both non-normalized and normalized libraries with Arabidopsis homologs were counted to be 6,737 and 2,187, respectively (Table 1). Distribution of these homologs in Arabidopsis covered all aspects of cellular components, molecular functions, and biological processes (Figure 5). The fold increase of genes in normalized versus non-normalized over different categories ranges from 1.3 (ribosome, abundant transcripts) to 6 (signal transduction, low abundant transcripts), averaging at 2.9, suggesting a higher efficiency in gene discovery using the normalization library. High levels of protein metabolism, cell organization and biogenesis, and transport in molecular process were also observed. Most genes in molecular function categories encode activities of hydrolase, transferase, and protein and nucleotide binding, activities typically found in the active expansion of embryo and oil biosynthesis.

The top 50 genes with the largest numbers of reads in the non-normalized library included primarily genes for seed storage proteins, including those related to napin, legumin, and 11 S globulin (Table 2). As an indication of the power of normalization for reducing the representation of these abundant genes, reads for transcripts of napin genes were reduced from 23,678 in the normalized library to 1,928 after normalization (Table 3). This resulted in a reduction in napin genes as the most abundant in numbers of reads in the non-normalized cDNAs to the fifth most abundant in the normalized cDNAs. Normalization also uncovered a number of genes that were not detected in the non-normalized library, including transcripts for genes encoding β-luffin, RPL24A, RPS17, PATL1, enoyl-reductase, and LRR proteins. Notably, β-luffin ranked number 3 in the normalized library with 2,110 reads, despite its absence from sequences in the non-normalized library.

**Table 3:** List of gene products for the 50 most abundant 454 transcript reads in the normalized cDNA library from bitter melon seeds.

	Normalized cDNAs				Non-normalized cDNAs	
Homolog ID	# of Reads	# of Contigs	At Gene Identifier	Gene Products	# of Reads	# of Contigs
gi|11225518	3,291	6	-	MAP30	8,197	45
gi|29243138	2,615	3	-	hypothetical protein	15,142	34
gi|19150	2,110	1	-	b-luffin	0	0
gi|13171073	1,701	1	-	ribosome-inactivating protein precursor	6,309	3
gi|21327881	1,928	12	-	napin [bitter melon charantia]	23,678	169
gi|3808062	632	1	At3g22640	cupin family protein	4,278	42
gi|29165641	618	1	-	trichosanthin precursor	1,804	5
gi|4106063	584	3	-	Trichoanguin	1,282	8
gi|147798373	767	4	At1g49320	BURP domain-containing protein	5,370	10
gi|157335527	414	2	At1g45201	similar to lipase class 3 family protein	1,000	3
gi|976231	460	2	At2g02990	ribonuclease (RNase LC1)	2,438	3
gi|170444	374	1	At2g02490	Extension (class II)	780	1
gi|157342180	274	1	At1g19100	ATP-binding region	41	1
gi|73808794	248	1	At4g09320	NDPK1 (nucleoside diphosphate kinase 1)	214	2
gi|18479082	238	1	At4g28520	11 S globulin-like protein	151	15
gi|84514139	231	1	At3g14610	CYP72A7; cytochrome P450	230	6
gi|157337377	230	1	At5g59750	riboflavin biosynthesis protein, putative	102	8
gi|157336060	219	1	At1g77460	C2 domain-containing protein	26	1
gi|118482397	202	1	At1g75270	DHAR2; glutathione dehydrogenase	833	3
gi|1838961	202	2	At4g25050	ACP4 (Acyl carrier protein 4)	714	2
gi|74229677	196	1	At1g08830	CSD1 (copper/zinc superoxide dismutase 1)	73	1
gi|162672646	170	1	-	predicted protein	0	0
gi|17402469	167	1	At4g14960	TUA6 (tubulin alpha-6 chiain)	46	2
gi|157350920	162	1	At1g07040	similar to unknown protein	12	2
gi|118481513	152	1	At2g36620	RPL24A	0	0
gi|157352507	142	1	At1g61580	ARP2/RPL3B	40	1
gi|157327297	156	2	At1g18540	60 S ribosomal protein L6 (RPL6A)	212	3
gi|118482623	131	1	At2g02130	LCR68/PDF2.3 (LMW cysteine-rich 68)	637	2
gi|37721219	127	1	AtCg00900	ribosomal protein S7	0	0
gi|77999293	157	2	At5g02960	40 S ribosomal protein S23 (RPS23B)	154	2
gi|145910320	126	1	At5g35360	acetyl co-enzyme A carboxylase subunit	24	3
gi|1762945	125	1	At4g14420	lesion inducing protein-related	222	1
gi|118484529	142	2	At3g53740	60 S ribosomal protein L36 (RPL36B)	266	4
gi|13430182	141	2	At3g04400	ribosomal protein L17	172	1
gi|157342677	123	1	At4g29520	similar to Saposin B	44	4
gi|21304463	121	1	AtCg01310	chloroplast ribosomal protein L2	80	2
gi|157344877	119	1	At1g72150	PATL1 (PATELLIN 1); transporter	0	0
gi|49617323	119	1	At3g27660	OLEO4 (OLEOSIN4)	28	1
gi|2204236	121	2	At2g05990	Enoyl-[acyl-carrier-protein] reductase	0	0
gi|20514369	118	1	At4g35160	O-methyltransferase family 2 protein	59	1
gi|118482052	118	1	At3g05560	structural constituent of ribosome	0	0
gi|157340503	129	2	At1g75760	ER lumen protein retaining receptor family	194	1
gi|157358487	138	2	At1g27330	similar to unknown membrane protein	1121	6
gi|71842524	137	3	At1g70580	AlaT1	97	1
gi|157339643	152	3	At5g55190	RAN3; GTP binding	188	4
gi|13899097	108	1	At5g21090	leucine-rich repeat protein, putative	0	0
gi|38093741	107	1	At1g31812	ACBP (ACYL-COA-BINDING PROTEIN)	4	1
gi|118482799	106	1	At1g08830	CSD1 (copper/zinc superoxide dismutase 1)	50	1
gi|157343130	150	2	At4g30810	SCPL29 (serine carboxypeptidase-like 29)	13	2
gi|1666094	103	1	At5g51810	gibberellin 20-oxidase	402	4

Numbers of reads and contigs and most related homolog for each gene product are also shown. For comparison, the numbers of read and contigs for each transcript in the non-normalized cDNA library are indicated.

As a first step toward understanding eleostearic acid biosynthesis and metabolism, we first attempted to identify all genes involved in lipid metabolism in developing bitter melon seeds. Normalized library sequences were compared with those in the annotated lipid metabolism gene database compiled at Michigan State University http://lipids.plantbiology.msu.edu/[33]. From this analysis, bitter melon cDNAs encoding enzymes for known lipid biosynthetic and catabolic pathways were identified. From this analysis, almost all categories of lipid metabolism, from synthesis of lipid in plastids and endomembranes, metabolism of acyl-lipids in mitochondria, synthesis and degradation of storage oil, lipid signaling, fatty acid elongation, wax and cutin metabolism, and a group of miscellaneous genes were identified (Table 1, Additional File 3). Among the genes not detected in either library were those for enzymes in lipid degradation pathways, including DAD1-like acylhydrolase, allene oxide cyclase, and patatin-like acyl-hydrolase. Transcripts for several other hydrolases, including wax ester hydrolase, fatty acid ω-hydrolase, and alcohol-forming fatty acyl coenzyme A reductase, were also not detected in the bitter melon seed transcriptome. Most of these enzymes are involved in the synthesis of surface lipids, such as waxes in leaves and flowers that are not present in the embryo. In total, we identified 345 bitter melon transcripts that share homology with 189 known lipid genes in Arabidopsis (Table 4). This information is provided in a searchable database (Additional File 4).

**Table 4:** Numbers of total transcript reads and contigs comprising different categories of lipid genes from 454 analysis of normalized cDNA from developing bitter melon seeds.

Categories	Numbers of reads	Numbers of contigs	Numbers of Arabidopsis homologs
Fatty acid synthesis in plastids	1,462	63	31
Synthesis of plastid membrane lipids	100	10	6
Synthesis of endomembrane lipids	674	59	31
Acyl-lipid metabolism in mitochondria	492	33	19
Synthesis and storage of oil	235	10	7
Storage lipid and fatty acid degradation	482	38	19
Lipid signaling	761	97	48
Fatty acid elongation & wax and cutin			
metabolism	108	7	5
Miscellaneous	845	78	43

Also shown are the numbers of unique homologs of known Arabidopsis genes in the assembled contigs. The categories are based on those used in the Arabidopsis Lipid Gene database [33].

### Transcriptomic analysis and gene mining for eleostearic acid metabolism in bitter melon seeds

**Figure 6:**
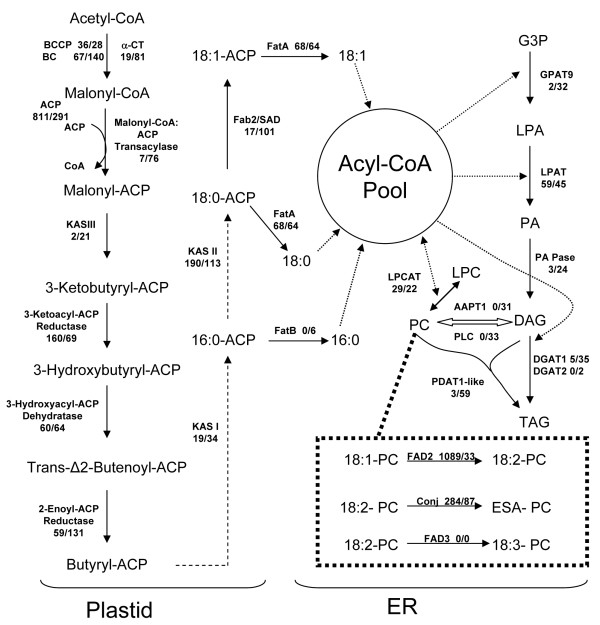
**Numbers of reads in the non-normalized and normalized 454 sequence analysis for genes encoding enzymes in the fatty acid and TAG biosynthetic pathways in bitter melon seeds**. Numbers after the gene (a/b) are the numbers of reads for the corresponding genes in the 454 analysis of cDNA libraries: a, non-normalized; b, normalized. The dashed arrows in the plastid fatty acid biosynthetic pathway indicate one or more cycles of acyl-chain elongation that is initiated by 3-ketoacyl-ACP synthase (KAS) I or II. Abbreviations: BCCP, biotin carboxyl carrier protein subunit of acetyl-CoA carboxylase; BC, biotin carboxylase subunit of acetyl-CoA carboxylase; α-CT, α-carboxyltransferase subunit of acetyl-CoA carboxylase; ACP, acyl carrier protein; FAB2/SAD, stearoyl-ACP desaturase; FatA, acyl-ACP thioesterase A; FatB, acyl-ACP thioesterase B; GPAT, glycerol 3-phosphate acyltransferase; LPAT, lysophosphatidic acid acyltransferase; PA Pase: phosphatidic acid phosphatase; LPCAT, lysophosphatidylcholine acyltransferase; AAPT, CDP-choline:diacyglyglycerol cholinephosphotransferase; PLC, phospholipase C-type enzymes; DGAT, diacylglycerol acyltransferase; PDAT, phospholipid:diacylglycerol acyltransferase; FAD2, Δ12 oleic acid desaturase; FAD3, Δ15 (ω-3) linoleic acid desaturase; G3P, glycerol-3-phosphate; LPA, lysophosphatidic acid; PA, phosphatidic acid; LPC, lysophosphatidylcholine; PC, phosphatidylcholine; DAG, diacylglycerol; TAG, triacylglycerol.

To gain a global perspective of lipid metabolism in bitter melon seeds, the abundance of 454 transcript reads for enzymes involved in fatty acid and TAG biosynthesis was compiled from 454 data obtained from non-normalized and normalized cDNA populations (Figure 6). With regard to *de novo *fatty acid synthesis in plastids, the largest numbers of reads in the non-normalized cDNAs were transcripts for acyl carrier protein (ACP, 811 reads), 3-keto-acyl-ACP synthase (KAS) II (190 reads) and 3-ketobutyl-ACP reductase (160 reads) (Figure 6). It is also notable that 68 reads were detected for the FatA acyl-ACP thioesterase, but no reads were detected for the FatB acyl-ACP thioesterase in the non-normalized cDNAs. This difference in numbers of reads and the known substrate preferences of Fat A (*i.e*., most active with 18:0- and 18:1-ACP) and Fat B (*i.e*., most active with 16:0-ACP) [34-36] likely account for the relatively high content of stearic acid (18:0) and low content of palmitic acid (16:0) in bitter melon seeds (Figure 1). Compared to *de novo *fatty acid synthesis, transcript reads for ER-associated lipid enzymes were of low abundance in the non-normalized cDNAs, except for FAD2 Δ12-oleic acid desaturases (1,089 reads) and fatty acid conjugase (284 reads) (Table 4, Figure 6).

For ER-associated TAG synthesis enzymes, normalization yielded significant enrichment of transcripts for enzymes including glycerol 3-phosphate acyltransferase 9 (GPAT9), phosphatidic acid phosphatase (PA Pase), diacylglycerol acyltransferase 1 (DGAT1), phospholipid:diacylglycerol acyltransferase 1 (PDAT1). Normalization also uncovered transcripts not detected in the non-normalized cDNA population, including those for diacylglycerol acyltransferase 2 (DGAT2), phospholipase C-type enzymes (PLC), CDP-choline:diacyglyglycerol cholinephosphotransferase (AAPT1). Notably, transcripts for the recently reported phosphatidylcholine:diacylglycerol cholinephosphotransferase (PDCT), a key enzyme in polyunsaturated fatty acid synthesis in Arabidopsis seeds [37], were not detected in either the non-normalized or normalized libraries. Similar to the findings reported here, no transcripts for PDCT were detected in a transcriptomic analysis of developing tung seeds, which also accumulate high levels of eleostearic acid (Shockey, unpublished data). These findings suggest that metabolic pathways independent of PDCT are associated with eleostearic acid metabolism. *FAD3 *transcripts for the Δ15 linoleic acid desaturase were also not detected in either cDNA library, which is consistent with the near absence of α-linolenic acid in developing bitter melon seeds.

**Figure 7:**
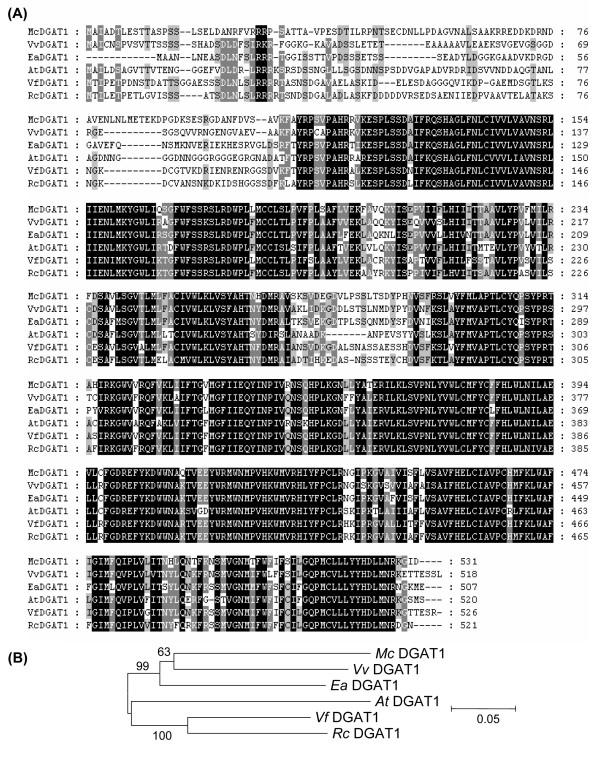
**Amino acid sequence alignments of McDGAT1 and DGAT1 polypeptides from various species**. (A) Alignment of various DGAT1 s. Sequences were aligned by Clustal × and displayed using GeneDoc Software. (B) Phylogenetic tree of DGAT1 polypeptides. Sequences of DGAT1 are from the following species: *Vitis vinifera (Vv) *(gi 225444869), *Euonymus alatus (Ea) *(gi 54145459), *Arabidopsis thaliana (At) *(gi15224779), *Vernicia fordii (Vf) *(gi 86279632), *Ricinus communis (Rc) *(gi 255546145). Phylogenetic tree was constructed using MEGA 4.0.1 software [52].

Eleostearic acid is synthesized by Δ12 conjugase activity on linoleic acid bound primarily to phosphatidylcholine [9]. Despite its site of synthesis, eleostearic acid is found nearly exclusively in TAG and accounts for < 2% of the fatty acids in phospholipids throughout the development of bitter melon seeds (Figure 1A). This implies that eleostearic acid is efficiently metabolized after its synthesis on phosphatidylcholine to its point of accumulation in TAG. To study the flux of eleostearic acid from its synthesis on phosphatidylcholine to storage in TAG, genes for lipid metabolism enzymes involved in fatty acid esterification and removal from glycerol backbones and those catalyzing removal or transfer of phospholipid head groups are of particular interest. Among these enzymes are the two classes of diacylglycerol acyltransferases: DGAT1 and DGAT2. These enzymes catalyze the esterification of fatty acids from acyl-CoA pools to the *sn*-3 position of DAG to form TAG. DGAT1 has been shown to be a major enzyme associated with TAG synthesis in Arabidopsis seeds [38,39]. In contrast, studies of DGAT2 T- DNA mutants have failed to identify a role for this enzyme in TAG accumulation in Arabidopsis seeds [38]. However, transgenic expression of DGAT2-type enzymes from castor bean and *Vernonia galamensis *have been shown to enhance the accumulation of ricinoleic acid in Arabidopsis seeds and vernolic acid in soybean seeds, respectively [40,41]. Results from tung also suggest that DGAT2 may be important in eleostearic acid metabolism in seeds of this species [42,43]. In the 454 sequence data, five reads for transcripts for DGAT1 and zero reads for DGAT2 were detected in the non-normalized cDNA library (Figure 6). Following normalization, 35 reads for DGAT1 transcripts and five reads for DGAT2 transcripts were detected (Figure 6). It is unclear whether the larger number of reads for DGAT1 transcripts indicates that this enzyme, rather than DGAT2, is of greater importance for eleostearic acid metabolism in bitter melon seeds. We are currently exploring this hypothesis by co-expression of bitter melon DGAT1 and DGAT2 with the bitter melon fatty acid conjugase in Arabidopsis conjugase to compare the relative abilities of these enzymes to enhance eleostearic acid accumulation. To facilitate these studies, full-length cDNAs for bitter melon DGAT1 and DGAT2 have been isolated. These sequences represent a single gene for each DGAT class. Alignments of the amino acid sequences of these enzymes with known DGAT1 and DGAT2 polypeptides are shown (Figure 7A and Additional File 5). The bitter melon DGAT1 is most closely related to the grape DGAT1 (70% identity) and *Euonymus alatus *DGAT1 (68% identity) but is more distantly related to DGAT1 enzymes from Arabidopsis (64% identity), castor (65% identity), and tung (66% identity) (Figure 7B). The bitter melon DGAT2 is most closely related to its homolog in Arabidopsis (57% identity), but distantly related to DGAT2 enzyme from castor (50% identity) and tung (49% identity). As shown in Figure 7A and Additional File 5, the N-terminal regions of the bitter melon DGATs and other known DGATs are the most variable portion of these polypeptides. One possibility is that the N-termini of DGATs are important determinants of the substrate specificities of these enzymes, especially with regard to the metabolism of unusual fatty acids.

In addition to DGATs, phospholipid:diacylglycerol acyltransferases (PDATs) are important enzymes for the final acylation step in TAG synthesis. In this regard, the activities of PDAT1 (At5g13640) and DGAT1 were recently shown to account for the bulk of TAG synthesis in Arabidopsis seeds [38]. Arabidopsis also contains a PDAT1-related gene (At3g44830) that displays seed-specific expression [44]. The polypeptide encoded by At3g44830 shares 57% amino acid sequence identity with PDAT1; however, the function of this polypeptide has yet to be established.

PDATs catalyze the transacylation of fatty acids from phospholipid to the *sn*-3 position of DAG and share homology with the well-studied enzyme lecithin:cholesterol acyltransferase (LCAT), which catalyzes sterol ester synthesis in blood plasma [45]. In plants, PDAT activity with high specificity for the transfer of ricinoleic was identified in microsomes of castor bean [46], suggesting the possibility that PDAT-type activity may also be an important contributor to eleostearic acid metabolism in bitter melon seeds. In our normalized cDNA library, two contigs (McCtg3028 and McCtg2714) were identified with closest relation to the PDAT1-like gene At3g44830. These two contigs were confirmed to one gene by PCR amplification, and this gene was designated *McPDAT1 *(data not shown). No close homologs for the At5g13640-encoded PDAT1 were detected in the non-normalized or normalized bitter melon sequence data. The role of McPDAT1 in eleostearic acid metabolism is currently being explored.

In addition to DGATs and PDATs, transcripts for numerous other enzymes that may be specialized for eleostearic acid metabolism were detected in the non-normalized and normalized libraries. These include transcripts for lysophosphatidic acid acyltransferasese (LPAT), phospholipase A_2_- and phospholipase C-related enzymes, and lysophosphatidylcholine acyltransferase (LPCAT).

### Identification of a FAD2 variant in developing bitter melon seeds

**Figure 8:**
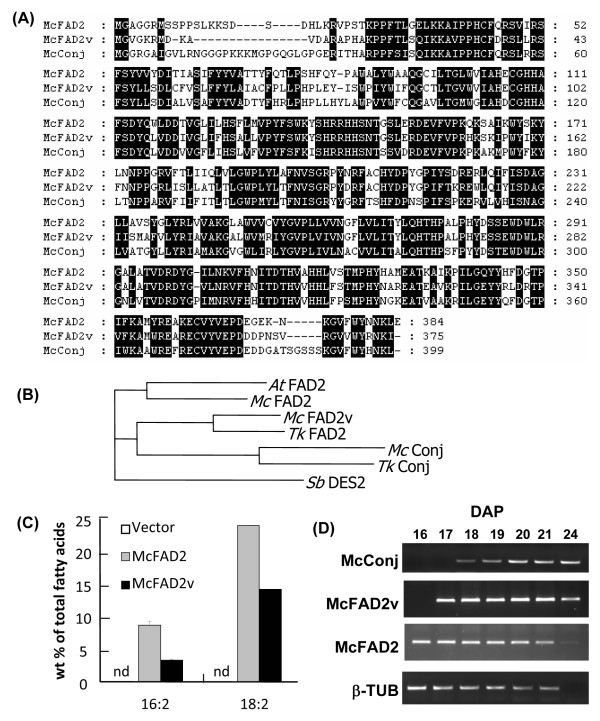
**Characterization of FAD2 polypeptides and genes in bitter melon**. (A) Alignment of amino sequences for divergent FAD2 s (McFAD2, McFAD2v) and the FAD2-related conjugase (McConj) from bitter melon cDNA library. Sequences were aligned by Clustal X and displayed using GeneDoc Software. (B). Phylogenetic tree of FAD2 s and conjugase. Sequences used to construct the tree include *Arabidopsis thaliana At *FAD2 (gi 21536781), *Trichosanthes kirilowii Tk *FAD2 (gi 28371823), *Tk *Conj (gi 28371821), and *Sorghum bicolor Sb *DES2 (gi 242062720). (C). Production of 16:2 and 18:2 in *Saccharomyces cerevisiae *by expression of McFAD2 and McFAD2v. As indicated, no 16:2 or 18:2 was detected in yeast cells containing only the vector. (D) Expression of McFAD2, McFAD2v, and conjugase (McConj) during bitter melon seed development as determined by RT-PCR. β-Tubulin (β-Tub) -specific primers were used as a control to assess the quality of first-strand cDNA.

An unexpected finding from the transcriptomic analysis of developing bitter melon seeds was the discovery of two divergent forms of FAD2, the Δ12 oleic acid desaturase. A detailed analysis of FAD2 contigs revealed two related but different sequences. One designated "McFAD2" was reported previously [10], and the second is an evolutionarily divergent FAD2 designated "McFAD2v" ("v" for variant). McFAD2 and McFAD2v share 69% amino acid sequence identity (Figure 8A). In addition, McFAD2 and McFAD2v share 60% and 63% amino acid sequence identity with the bitter melon conjugase, respectively. McFAD2v is most closely related to a variant FAD2 identified in seeds of snake gourd (*Trichosanthes kirilowii*) that synthesizes the conjugated fatty acid punicic acid (18:3 Δ^9*cis*,11*trans*,13*cis*^) (Figure 8B) [12]. Although it is more distantly related to McFAD2, McFAD2v lacks amino acid substitutions in the proximity of the catalytic His box domains that are characteristic of functionally variant FAD2-type enzymes (e.g., conjugase, epoxygenase, hydroxylase) [47], suggesting that McFAD2v is likely a typical Δ12 oleic acid desaturase (Figure 8A).

To establish the functions of McFAD2 and McFAD2v, the open-reading frames of these enzymes were assembled under control of the *GAL10 *promoter in the pESC-URA vector and expressed in *Saccharomyces cerevisiae*. In galactose-induced cultures for both desaturases, production of 16:2 and 18:2 were detectable (Figure 8C). Neither fatty acid was detected in induced cultures containing the pESC-URA vector lacking cDNA insert. In addition, no conjugated fatty acids were detected in the induced McFAD2 or McFAD2v cultures. As a control, the bitter melon conjugase was also expressed in *S. cerevisiae*. Unlike McFAD2 and McFAD2v, the conjugase displayed mixed functionality; generating small amounts of 16:2 and 18:2 from Δ12 desaturase activity as well as eleostearic acid from conjugase activity with 18:2 (data not shown). These results indicate that both McFAD2 and McFAD2v function as Δ12 oleic desaturases despite their divergent sequences.

Gene expression studies were conducted to understand the basis for two functional Δ12 oleic acid desaturases in developing bitter melon seeds. Using RT-PCR, expression profiles of genes for McFAD2, McFAD2v, and the conjugase were obtained during seed development (Figure 8D). Interestingly, expression of the conjugase gene most closely mirrored the timing for expression of *McFAD2v *during seed development. By comparison, expression of *McFAD2 *was detected earlier in seed development. Given the similarity in their gene expression patterns, McFAD2v may have evolved to function in concert with the conjugase for eleostearic acid synthesis in bitter melon seeds. In this regard, the Δ12 oleic acid desaturase provides the linoleic acid substrate for production of eleostearic acid by the conjugase. It is notable that transgenic expression of the bitter melon conjugase in Arabidopsis seeds and soybean somatic embryos results in large increases in the relative content of oleic acid, in a manner consistent with the apparent inhibition of native Δ12 oleic acid desaturase activity [9,10]. For example, relative amounts of oleic acid in seeds of non-transformed Arabidopsis Col-0 *fad3/fae1 *increase from ~28% of the total fatty acids to nearly 55% of the total fatty acids in seeds that express the bitter melon conjugase [9]. Although a number of biochemical scenarios could be proposed, one possibility for future study is that McFAD2v and the conjugase functionally interact to maintain efficient synthesis of eleostearic acid in bitter melon seeds. The role of two FAD2-related enzymes in the synthesis of an unusual fatty acid has previously been demonstrated in the synthesis of dimorphecolic acid in *Dimorphotheca sinuata *seeds [15].

## Conclusions

Deep sequencing of developing bitter melon seeds was conducted to identify candidate genes that are associated with the synthesis of the conjugated fatty acid eleostearic acid and the efficient metabolism of eleostearic acid from its synthesis on phosphatidylcholine to storage in TAG. By use of 454 pyrosequencing of non-normalized cDNAs derived from bitter melon seeds at 18 DAP, 190 contigs with homology to 83 known lipid genes in Arabidopsis were obtained from 10,072 total contigs. The discovery of lipid genes was significantly enhanced through the normalization of cDNAs based on the use of duplex-specific nuclease. 454 sequence data from a normalized library generated 345 contigs with homology to 189 known lipid genes in Arabidopsis from 18,245 total contigs, although the total number of clean reads from the normalized library was 22% lower than that obtained from the non-normalized library. Overall, transcriptomic analysis of bitter melon seeds using 454 technology yielded sequence data for genes encoding all of the known fatty acid biosynthetic enzymes and nearly all of the known ER-associated fatty acid modification and metabolic enzymes, including acyltransferases such as DGAT1, DGAT2, and a PDAT1-related enzyme that are likely central to efficient metabolism of eleostearic acid. Also identified in the transcriptomic analysis was a divergent FAD2 that was demonstrated to have Δ12-oleic acid desaturase activity and may be important in the synthesis of eleostearic acid. The sequence information from developing bitter melon seeds has been made publicly available in a searchable format http://genomics.msu.edu/JO/blast/blast.html; Additional File 4) and will likely serve as a useful resource for studies of unusual fatty acid metabolism in plants and for engineering of conjugated fatty acid production in oilseed crops.

## Methods

### Growth conditions of plants and collection of seeds

*Momordica charantia *L. was grown under short-day conditions with 8 h light at 25°C/16 h dark at 21°C, 50% humidity, and 600 μmol m^-1 ^s^-1 ^of light. Independent male flowers were used for hand pollination of female flowers. Embryos were dissected from seeds of fruits collected at specific DAP and frozen immediately in liquid nitrogen. Embryos were stored at - 80°C until use in RNA isolation or lipid analysis.

### Lipid analysis of bitter melon embryos

Total lipids were extracted from frozen bitter melon embryos as described [9] using a modified version of the method reported by Bligh and Dyer [48]. Neutral lipids (consisting predominantly of TAGs), glycolipids, and phospholipids were partitioned from the total lipids by solid phase extraction (SPE) using commercially prepared silica columns (500 mg silica bed; Fisher Scientific). The total lipid extract was dissolved in one ml of chloroform and applied to a SPE column that had been equilibrated in chloroform. Neutral lipids were eluted with ten ml of chloroform and five ml of chloroform:acetone (80:20 v/v). Glycolipids were then eluted with seven ml of acetone. Phospholipids were subsequently eluted with five ml of methanol:chloroform:water (100:50:40 v/v/v). To the phospholipid fraction, 1.3 ml of water and 1.3 ml of chloroform were added. After mixing and centrifugation, the lower organic phase containing phospholipids was recovered. The neutral and phospholipid fractions in glass screw cap test tubes (13 × 100 mm) were dried under nitrogen and then transesterified with the addition of 1.5 ml of 1% sodium methoxide in methanol (w/v) and 0.2 ml of toluene. For quantification of fatty acids, triheptadecanoin (Nu-Chek, Elysian, Minnesota USA) was also added to each fraction as an internal standard. Transesterification and subsequent recovery and analysis of fatty acid methyl esters by gas chromatography was conducted as previously described [9].

### RNA extraction and RT-PCR analysis

RNA was extracted from bitter melon seeds using the Trizol reagent as described by the manufacturer (Invitrogen). RT-PCR was carried out using the Advantage RT-for-PCR kit from BD Biosciences Clontech. In brief, 1 μg of total RNA was reverse transcribed, and the cDNA was used in PCR reactions to amplify the corresponding genes with FAD2- or conjugase, oleosin, or β-Tubulin-specific primers, respectively. The conjugase primers were 5'-McConj-1 (5' CTCCCTTCAGCATCAGCCAG -3') and 3'-McConj-1 (5'-TACAGCAAATACCCGGTCGC-3'); the β-Tubulin primers were 5'-McTUB2-1 (5'-AGATCGGTGCCAAGTTCTGG-3') and 3'-McTUB2-1 (5'-GATGGACAGAGAGGGTTGCG-3'), and oleosin primers were 5'-McC58-1 (5'-ATGGCCGAGCACCAGCAG-3') and 3'-McC58-1 (TTAAGAAGTTGCAGTCTGGGGG-3'). The FAD2v primers were 5'-McFAD2v-1 (5'-ATGGACAAGGCCGTTGATGC-3') and 3'-McFAD2v-2 (5'-GTAGAGCACGAAAGCCATGCTG-3'), and FAD2 primers were 5'-McFAD2-1 (5'-TCCTCTCCTCCATCCCTCAAG-3') and 3'-McFAD2-1 (5'-CGTACGAGACGGCCAGCAAC-3'). PCR was conducted for number of cycles as indicated in the figures, with a primer-annealing temperature of 50°C. PCR products were analyzed by gel electrophoresis.

### cDNA library construction and normalization

Total RNA was extracted from developing bitter melon seeds was ground to a fine powder in liquid nitrogen using Trizol reagent (Invitrogen) according too the manufacturer's protocol. mRNA were purified from ~1 mg of total RNA by two passes through oligo-dT cellulose columns by use of the Illustra mRNA purification kit (GE Healthcare). cDNA libraries were constructed using SMART PCR cDNA synthesis kit (Clontech). First-strand cDNA was synthesized with 150 ng mRNA in a volume of 10 μl using the provided SMART II primer, a modified CDS III/3' cDNA synthesis primer (5'-AAGCAGTGGTATCAACGCAGAGTGGCCGAGGCGGCCGACATGTTTTGTTTTTTTTTC TTTTTTTTTTVN-3') and Superscript II reverse transcriptase (Invitrogen). Double stranded cDNA was prepared by PCR (18 cycles) using 2 μl of the first-strand reaction in a 50 μl reaction volume. Following Proteinase K treatment, four PCR reactions were pooled before SfiI digestion and size fractionated on the provided CHROMA SPIN-400 column. Only fractions containing fragments larger than 500 bp were collected, precipitated, and resuspended in TE buffer. Library normalization of this cDNA was conducted by use of Trimmer-Direct cDNA normalization kit (Evrogen). Briefly, four 250 ng aliquots of cDNA were hybridized at 98°C for 2 min followed by 68°C for 5 h. The hybridized cDNAs were then treated with 0, 0.25, 0.5, and 1 μl duplex-specific nuclease (DSN), respectively, before stop with the DSN stop buffer. cDNA (1 μl) from each aliquot was subjected to PCR amplification. Based on the results from the sample lacking DSN, the cycle number (9+2 cycles) was determined for the first round amplification of DSN treated samples. After examination of the cDNAs on the agarose gel, the selected aliquot of cDNAs were then diluted 10 times and subjected to a second round of PCR using 2 μl in 100 μl reaction (12 cycles). The amplified cDNA pool was then treated with proteinase K, fractionated, and precipitated for the non-normalized cDNA library construction. For the pilot study, cDNA PCR fragments were digested with *Sfi*I enzyme and cloned into *Sfi*IA and *Sfi*IB sites of pDNR-LIB vector (Clontech).

### 454 sequencing and data analysis

DNA sequencing was performed at the Michigan State University Research Technology Support Facility using the GS FLX sequencer (Roche). Reads were trimmed to remove low quality and primer sequences using Seq- Clean [49]. The reduced dataset then underwent two rounds of assembly with CAP3. First-round CAP3 parameter settings for percent match, overlap length, maximum overhang percent, gap penalty, and base quality cutoff for clipping were -p 90 -o 50 -h 15 -g 2 -c 17, respectively. For the second round, -o was changed to 100. The resultant contigs were then annotated with a translated BLAST against the TAIR7 and the viridiplantae subdivision of the NCBI nonredundant protein databases.

Sequence data have been deposited in the GenBank Short Read Archive (SRA). The accession number for the project in NCBI SRA is SRP004091. The accession numbers in NCBI SRA for the individual experiments are SRX030203 (normalized sequence data) and SRX030204 (non-normalized sequence data). The assembled sequence data are also available in a searchable format at http://genomics.msu.edu/JO/blast/blast.html, and lipid gene data are compiled in Additional File 4.

### Sequence alignment and phylogenetic analysis

Protein sequences were aligned using the clustal W Multiple Sequence Alignment Program [50] using Gonnet protein weight matrix (gap open penalty = 10, gap extension penalty = 0.2, gap separation distance = 4) and displayed by GeneDoc [51]. Phylogenetic trees of protein sequences (aligned with Clustal W) was generated in MEGA4.0.1 [52] using the neighbor-joining method [53]. Pairwise deletion was used for handling of sequence gaps, and 2000 bootstrap replicates were performed. The evolutionary distances were computed using the Poisson correction method [54].

### Functional analysis of McFAD2 and McFAD2v genes

McFAD2 and McFAD2V were expressed in *S. cerevisiae *using pESC-URA vector (Stratagene), which contains separate GAL10 promoters for expression. Open reading frames for McFAD2 and McFAD2V were amplified by PCR from a bitter melon cDNA library using Phusion polymerase (New England Biolabs). PCR products were digested with *Spe*I/*Pac*I before and cloned under control of the *GAL10 *promoter into the corresponding restriction sites in pESC-URA. The oligonucleotides used for PCR were: 5'- McFAD2_ SpeI (5' ATATACTAGTATGGGTGCTGGAGGCCGAAT 3'), 3'- McFAD2_ PacI (5' ATATTTAATTAATTATTCCAACTTGTTGTTGT 3'), McFAD2v_ SpeI (5' ATATACTAGTATGGGAGTTGGAAAAAGAAT 3'), 3'- McFAD2v_ PacI (5' TTAATTAATTAATCAGATCTTGTTGCGGTACCA 3'). (Note that the underlined sequences correspond to the added corresponding restriction sites.) These pESC-URA-derived plasmids were transformed into *S. cerevisiae *strain YPH499, and expression studies and fatty acid analyses of induced cells were conducted as described [15].

## List of Abbreviations

Abbreviations: AAPT: CDP-choline:diacyglyglycerol cholinephosphotransferase; ACP: acyl carrier protein; BC: biotin carboxylase subunit of acetyl-CoA carboxylase; BCCP: biotin carboxyl carrier protein subunit of acetyl-CoA carboxylase; α-CT: α-carboxyltransferase subunit of acetyl-CoA carboxylase; DAG: diacylglycerol; DAP: days after pollination; FAB2/SAD: stearoyl-ACP desaturase; DGAT: diacylglycerol acyltransferase; ESA: eleostearic acid; FAD2: Δ12 oleic acid desaturase; FAD3: Δ15 (ω-3) linoleic acid desaturase; FatA: acyl-ACP thioesterase A; FatB: acyl-ACP thioesterase B; G3P: glycerol-3-phosphate; GPAT: glycerol 3-phosphate acyltransferase; LPA: lysophosphatidic acid; LPAT: lysophosphatidic acid acyltransferase; LPC: lysophosphatidylcholine; LPCAT: lysophosphatidylcholine acyltransferase; PLC: phospholipase C-type enzymes; PA: phosphatidic acid; PA Pase: phosphatidic acid phosphatase; PC: phosphatidylcholine; PDAT: phospholipid:diacylglycerol acyltransferase; RT-PCR: reverse transcription-polymerase chain reaction; SPE: solid phase extraction; TAG: triacylglycerol.

## Authors' contributions

PY, XL, and EBC designed research; PY, MJS, JMS, and XL performed research; PY and XL analyzed data; PY and EBC wrote the paper. All authors read and approved the final manuscript.

## Supplementary Material

Additional File  1 **Pilot sequencing results of independent colonies from non-normalized and normalized cDNA libraries prepared from bitter melon seeds collected at 18 DAP**. Click here for file

Additional File  2 **The most abundant contigs identified by 454 sequencing of non-normalized cDNAs from developing bitter melon seeds**. Click here for file

Additional File  3 **Arabidopsis lipid genes with no detected homolog in 454 sequences from bitter melon seeds**. Click here for file

Additional File  4 **Bitter melon lipid gene database from 454 sequencing of normalized cDNA populations**. Click here for file

Additional File  5 **Alignment of McDGAT2 and DGAT2 polypeptides from other plant species**. Click here for file
