# Supplementary material for: Mining the bitter melon (momordica charantia l.) seed transcriptome by 454 analysis of non-normalized and normalized cDNA populations for conjugated fatty acid metabolism-related genes
Source: BMC Plant Biol. 2010 Nov 16;10:250. doi: 10.1186/1471-2229-10-250 (PMC3012625; doi:10.1186/1471-2229-10-250)
Supplement: Additional File 1 — Pilot sequencing results of independent colonies from non-normalized and normalized cDNA libraries prepared from bitter melon seeds collected at 18 DAP. [file 1471-2229-10-250-S1.DOC]

Additional File 1. Pilot sequencing results of independent colonies from non-normalized and normalized cDNA libraries prepared from bitter melon seeds collected at 18 DAP. Number of clones with the corresponding sequence is one unless indicated.

**Colonies from Non-normalized cDNA library**

Napin 4 clones

CLE14 (CLAVATA3/ESR-RELATED 14) 2 clones

ATP synthase delta chain

Trypsin inhibitor 2 clones

Unknown protein product

CAD80093.1 hypothetical protein 4 clones

Homologous to hypothetical protein *Vitis vinifera*

LOC100247554 Elastase inhibitor IV 2 clones

**Colonies from Normalized cDNA library**

Stearoyl-ACP desaturase

eIF4G

PHD finger protein

RHD3 (root hair defective 3 GTP-binding) protein

Cornichon family protein

Protein transporter sec61

Cp10, chaperonin21

2-phosphoglycerate kinase

Trypsin inhibitor 2 clones

Plastid serine hydroxymethyltransferase

Primer self ligated

Ribosome-inactivating protein momordin I precursor

AAA-ATPase vascular sorting VPS4 family

60S ribosomal protein L4/L1 (RPL4A)

Mitochondrial pyruvate dehydrogenase kinase isoform 2

Phosphoribosylformimino-5-aminoimidazole

carboxamide ribonucleotide isomerase

Transducin family protein / WD-40 repeat family protein

Ribosome-inactivating protein 2 clones

UDP-glucuronate decarboxylase 1

Dihydrodipicolinate reductase

AT-hook, DNA-binding protein

Unknown protein (*Vitis vinifera*)

Short match armadillo repeat protein

Fumarylacetoacetate hydrolase
