# Supplementary material for: Mining the bitter melon (momordica charantia l.) seed transcriptome by 454 analysis of non-normalized and normalized cDNA populations for conjugated fatty acid metabolism-related genes
Source: BMC Plant Biol. 2010 Nov 16;10:250. doi: 10.1186/1471-2229-10-250 (PMC3012625; doi:10.1186/1471-2229-10-250)
Supplement: Additional File 2 — The most abundant contigs identified by 454 sequencing of non-normalized cDNAs from developing bitter melon seeds. [file 1471-2229-10-250-S2.DOC]

Additional File 2. The most abundant contigs identified by 454 sequencing of non-normalized cDNAs from developing bitter melon seeds.

| **Contig ID** | **# of Reads** | **Length**  **(nt)** | **Viridiplant homolog** | **Functional description of gene product** |
| --- | --- | --- | --- | --- |
| [Mco2_320](http://genomics.msu.edu/cgi-bin/blasttools/seq_retrieve.pl?file=Mco2.fasta&key=Mco2_320) | 10,212 | 839 | gi|21327881 | napin - bitter melon |
| [Mco2_53](http://genomics.msu.edu/cgi-bin/blasttools/seq_retrieve.pl?file=Mco2.fasta&key=Mco2_53) | 8,248 | 674 | gi|29243138 | hypothetical protein - bitter melon |
| [Mco2_567](http://genomics.msu.edu/cgi-bin/blasttools/seq_retrieve.pl?file=Mco2.fasta&key=Mco2_567) | 7,573 | 1,001 | gi|11225518 | MAP30 - bitter melon |
| [Mco2_5](http://genomics.msu.edu/cgi-bin/blasttools/seq_retrieve.pl?file=Mco2.fasta&key=Mco2_5) | 7,451 | 2,038 | gi|56788031 | seed storage protein |
| [Mco2_38](http://genomics.msu.edu/cgi-bin/blasttools/seq_retrieve.pl?file=Mco2.fasta&key=Mco2_38) | 6,795 | 1,034 | - |  |
| [Mco2_661](http://genomics.msu.edu/cgi-bin/blasttools/seq_retrieve.pl?file=Mco2.fasta&key=Mco2_661) | 6,304 | 1,851 | gi|13171073 | ribosome-inactivating protein precursor |
| [Mco2_691](http://genomics.msu.edu/cgi-bin/blasttools/seq_retrieve.pl?file=Mco2.fasta&key=Mco2_691) | 6,048 | 1,734 | gi|167492 | 11-S globulin beta-subunit precursor |
| [Mco2_935](http://genomics.msu.edu/cgi-bin/blasttools/seq_retrieve.pl?file=Mco2.fasta&key=Mco2_935) | 5,215 | 766 | - |  |
| [Mco2_58](http://genomics.msu.edu/cgi-bin/blasttools/seq_retrieve.pl?file=Mco2.fasta&key=Mco2_58) | 5,145 | 1,387 | gi|147798373 | hypothetical protein |
| [Mco2_543](http://genomics.msu.edu/cgi-bin/blasttools/seq_retrieve.pl?file=Mco2.fasta&key=Mco2_543) | 4,178 | 745 | gi|21327881 | napin - bitter melon |
| [Mco2_162](http://genomics.msu.edu/cgi-bin/blasttools/seq_retrieve.pl?file=Mco2.fasta&key=Mco2_162) | 3,804 | 1,176 | gi|29243138 | hypothetical protein - bitter melon |
| [Mco2_106](http://genomics.msu.edu/cgi-bin/blasttools/seq_retrieve.pl?file=Mco2.fasta&key=Mco2_106) | 3,523 | 596 | - |  |
| [Mco2_746](http://genomics.msu.edu/cgi-bin/blasttools/seq_retrieve.pl?file=Mco2.fasta&key=Mco2_746) | 3,251 | 698 | gi|19528 | α-momorcharin - bitter melon |
| [Mco2_778](http://genomics.msu.edu/cgi-bin/blasttools/seq_retrieve.pl?file=Mco2.fasta&key=Mco2_778) | 3,192 | 547 | - |  |
| [Mco2_791](http://genomics.msu.edu/cgi-bin/blasttools/seq_retrieve.pl?file=Mco2.fasta&key=Mco2_791) | 3,135 | 821 | gi|19528 | α -momorcharin - bitter melon |
| [Mco2_590](http://genomics.msu.edu/cgi-bin/blasttools/seq_retrieve.pl?file=Mco2.fasta&key=Mco2_590) | 2,987 | 535 | - |  |
| [Mco2_722](http://genomics.msu.edu/cgi-bin/blasttools/seq_retrieve.pl?file=Mco2.fasta&key=Mco2_722) | 2,983 | 1,296 | gi|3808062 | PV100 cupin family protein |
| [Mco2_898](http://genomics.msu.edu/cgi-bin/blasttools/seq_retrieve.pl?file=Mco2.fasta&key=Mco2_898) | 2,490 | 1,158 | - |  |
| [Mco2_425](http://genomics.msu.edu/cgi-bin/blasttools/seq_retrieve.pl?file=Mco2.fasta&key=Mco2_425) | 2,401 | 588 | - |  |
| [Mco2_490](http://genomics.msu.edu/cgi-bin/blasttools/seq_retrieve.pl?file=Mco2.fasta&key=Mco2_490) | 2,108 | 754 | gi|29243138 | hypothetical protein - bitter melon |
| [Mco2_408](http://genomics.msu.edu/cgi-bin/blasttools/seq_retrieve.pl?file=Mco2.fasta&key=Mco2_408) | 2,107 | 909 | gi|976231 | ribonuclease (RNase LC1) |
| [Mco2_3811](http://genomics.msu.edu/cgi-bin/blasttools/seq_retrieve.pl?file=Mco2.fasta&key=Mco2_3811) | 1,916 | 377 | - |  |
| [Mco2_760](http://genomics.msu.edu/cgi-bin/blasttools/seq_retrieve.pl?file=Mco2.fasta&key=Mco2_760) | 1,895 | 1,020 | gi|157343662 | unnamed protein product |
| [Mco2_244](http://genomics.msu.edu/cgi-bin/blasttools/seq_retrieve.pl?file=Mco2.fasta&key=Mco2_244) | 1,844 | 665 | gi|21327881 | napin - bitter melon |
| [Mco2_163](http://genomics.msu.edu/cgi-bin/blasttools/seq_retrieve.pl?file=Mco2.fasta&key=Mco2_163) | 1,797 | 441 | - |  |
| [Mco2_441](http://genomics.msu.edu/cgi-bin/blasttools/seq_retrieve.pl?file=Mco2.fasta&key=Mco2_441) | 1,795 | 619 | - |  |
| [Mco2_68](http://genomics.msu.edu/cgi-bin/blasttools/seq_retrieve.pl?file=Mco2.fasta&key=Mco2_68) | 1,791 | 1,094 | gi|29165641 | trichosanthin precursor |
| [Mco2_328](http://genomics.msu.edu/cgi-bin/blasttools/seq_retrieve.pl?file=Mco2.fasta&key=Mco2_328) | 1,610 | 510 | gi|109895116 | ribosome-inactivating protein |
| [Mco2_991](http://genomics.msu.edu/cgi-bin/blasttools/seq_retrieve.pl?file=Mco2.fasta&key=Mco2_991) | 1,573 | 565 | - |  |
| [Mco2_497](http://genomics.msu.edu/cgi-bin/blasttools/seq_retrieve.pl?file=Mco2.fasta&key=Mco2_497) | 1,515 | 659 | - |  |
| [Mco2_774](http://genomics.msu.edu/cgi-bin/blasttools/seq_retrieve.pl?file=Mco2.fasta&key=Mco2_774) | 1,463 | 1,983 | gi|15215752 | riboflavin biosynthesis protein, putative |
| [Mco2_77](http://genomics.msu.edu/cgi-bin/blasttools/seq_retrieve.pl?file=Mco2.fasta&key=Mco2_77) | 1,299 | 766 | - |  |
| [Mco2_49](http://genomics.msu.edu/cgi-bin/blasttools/seq_retrieve.pl?file=Mco2.fasta&key=Mco2_49) | 1,298 | 470 | - |  |
| [Mco2_553](http://genomics.msu.edu/cgi-bin/blasttools/seq_retrieve.pl?file=Mco2.fasta&key=Mco2_553) | 1,294 | 1,706 | gi|5381325 | 11S globulin precursor |
| [Mco2_388](http://genomics.msu.edu/cgi-bin/blasttools/seq_retrieve.pl?file=Mco2.fasta&key=Mco2_388) | 1,264 | 1,143 | gi|4106063 | trichoanguin |
| [Mco2_392](http://genomics.msu.edu/cgi-bin/blasttools/seq_retrieve.pl?file=Mco2.fasta&key=Mco2_392) | 1,145 | 861 | gi|3808062 | PV100 cupin family protein |
| [Mco2_997](http://genomics.msu.edu/cgi-bin/blasttools/seq_retrieve.pl?file=Mco2.fasta&key=Mco2_997) | 1,103 | 766 | gi|21327881 | napin - bitter melon |
| [Mco2_9](http://genomics.msu.edu/cgi-bin/blasttools/seq_retrieve.pl?file=Mco2.fasta&key=Mco2_9) | 1,084 | 650 | gi|157358487 | unnamed protein product |
| [Mco2_899](http://genomics.msu.edu/cgi-bin/blasttools/seq_retrieve.pl?file=Mco2.fasta&key=Mco2_899) | 1,003 | 735 | gi|21327881 | napin - bitter melon |
| [Mco2_298](http://genomics.msu.edu/cgi-bin/blasttools/seq_retrieve.pl?file=Mco2.fasta&key=Mco2_298) | 990 | 1,543 | gi|157335527 | similar to lipase class 3 family protein |
| [Mco2_103](http://genomics.msu.edu/cgi-bin/blasttools/seq_retrieve.pl?file=Mco2.fasta&key=Mco2_103) | 964 | 648 | gi|21327881 | napin - bitter melon |
| [Mco2_537](http://genomics.msu.edu/cgi-bin/blasttools/seq_retrieve.pl?file=Mco2.fasta&key=Mco2_537) | 964 | 495 | - |  |
| [Mco2_842](http://genomics.msu.edu/cgi-bin/blasttools/seq_retrieve.pl?file=Mco2.fasta&key=Mco2_842) | 910 | 476 | - |  |
| [Mco2_585](http://genomics.msu.edu/cgi-bin/blasttools/seq_retrieve.pl?file=Mco2.fasta&key=Mco2_585) | 826 | 964 | gi|118482397 | unknown |
| [Mco2_90](http://genomics.msu.edu/cgi-bin/blasttools/seq_retrieve.pl?file=Mco2.fasta&key=Mco2_90) | 820 | 819 | - |  |
| [Mco2_255](http://genomics.msu.edu/cgi-bin/blasttools/seq_retrieve.pl?file=Mco2.fasta&key=Mco2_255) | 780 | 1,052 | gi|170444 | extensin (class II) |
| [Mco2_521](http://genomics.msu.edu/cgi-bin/blasttools/seq_retrieve.pl?file=Mco2.fasta&key=Mco2_521) | 705 | 305 | - |  |
| [Mco2_448](http://genomics.msu.edu/cgi-bin/blasttools/seq_retrieve.pl?file=Mco2.fasta&key=Mco2_448) | 671 | 728 | gi|1838961 | acyl carrier protein |
| [Mco2_631](http://genomics.msu.edu/cgi-bin/blasttools/seq_retrieve.pl?file=Mco2.fasta&key=Mco2_631) | 669 | 727 | - |  |
| [Mco2_687](http://genomics.msu.edu/cgi-bin/blasttools/seq_retrieve.pl?file=Mco2.fasta&key=Mco2_687) | 636 | 699 | gi|21327881 | napin - bitter melon |
