# Supplementary material for: Mining the bitter melon (momordica charantia l.) seed transcriptome by 454 analysis of non-normalized and normalized cDNA populations for conjugated fatty acid metabolism-related genes
Source: BMC Plant Biol. 2010 Nov 16;10:250. doi: 10.1186/1471-2229-10-250 (PMC3012625; doi:10.1186/1471-2229-10-250)
Supplement: Additional File 3 — Arabidopsis lipid genes with no detected homolog in 454 sequences from bitter melon seeds. [file 1471-2229-10-250-S3.DOC]

Additional File 3. Arabidopsis lipid genes with no detected homolog in 454 sequences from bitter melon seeds.

| **Classification of functions** | **Genes** |
| --- | --- |
| **Synthesis of fatty acids in plastids** | Plastidial Homomeric Acetyl-CoA Carboxylase  Plastidial Lipoate Synthase  Plastidial Holo-ACP Synthase |
| **Synthesis of membrane lipids in plastids** | Plastidial Phosphatidic Acid Phosphatase  Monogalactosyldiacylglycerol Synthase  Digalactosyldiacylglycerol Synthase  Sulfolipid Synthase  Plastidial Glycerol-Phosphate Acyltransferase  Plastidial 1-acylglycerol-Phosphate Acyltransferase  Plastidial Linoleate Desaturase (FAD7/FAD8)  Permease-like Protein of Outer Chloroplast Envelope |
| **Synthesis of membrane lipids in endomembrane system** | Ethanolamine Kinase  Phosphatidylserine Decarboxylase  ER Glycerol-Phosphate Acyltransferase  Ceramide Sphingobase Δ8 Desaturase  Glucosylceramide Synthase (Sterol Glucoside : Ceramide glucosyltransferase)  Ceramide Sphingobase Δ4 Desaturase  ER Phosphatidylglycerophosphate Synthase |
| **Metabolism of acyl-lipids in mitochondria** | Mitochondrial Ketoacyl-ACP Synthase  Mitochondrial 2-Lysophosphatidate Acyltransferase  Mitochondrial Phosphatidate Phosphatase  Mitochondrial Ketoacyl-ACP Reductase  Mitochondrial Lipoyltransferase  Mitochondrial Glycerol-3-Phosphate Dehydrogenase |
| **Degradation of storage lipids and straight fatty acids** | Peroxisomal Long-Chain Acyl-CoA Synthetase  Peroxisomal Enoyl-CoA Hydratase |
| **Lipid signaling** | Secretory Phospholipase A2  DAD1-like Acylhydrolase  Allene Oxide Cyclase  Hydroperoxide Lyase  α-Dioxygenase-Peroxidase (involved in FA α-oxidation)  Myotubularin-like Phosphoinositide 3-Phosphatase  Patatin-like Acyl-Hydrolase  Diacylglycerol Pyrophosphate Phosphatase  Phosphatidylinositol-4-Kinase β  GPI-specific Phospholipase C  Other Phospholipase D gamma |
| **Fatty acid elongation and wax and cutin metabolism** | Fatty Acid Omega-Hydroxylase  Alcohol-forming Fatty Acyl-Coenzyme A Reductase  Wax Synthase  CER1 Protein involved in wax synthesis  Putative transcription factor CER2 involved in wax biosynthesis  CER3 Protein involved in wax biosynthesis  ELO-like Elongase (activity not documented in plants)  Bifunctional wax ester synthase / DAGAT (activity not documented in plants) |
| **Miscellaneous** | Plastid Lipid-associated Protein  Plastidial Long-Chain Acyl-CoA Synthetase  Plastidial ABC Acyl Transporter  Lipid Transfer Protein type 1  Lipid Transfer Protein type 3  Lipid Transfer Protein type 4  Lipid Transfer Protein type 6  Lipid Transfer Protein type 7  Lipid Transfer Protein type 8  Acyl-CoA Desaturase-like  Pollen-surface Oleosin  Malonyl-CoA Decarboxylase  Cyclopropane Fatty Acid Synthase (activity not documented in Arabidopsis)  PPT1-like Thioesterase (activity not documented in plants) |
