# Supplementary material for: Mining the bitter melon (momordica charantia l.) seed transcriptome by 454 analysis of non-normalized and normalized cDNA populations for conjugated fatty acid metabolism-related genes
Source: BMC Plant Biol. 2010 Nov 16;10:250. doi: 10.1186/1471-2229-10-250 (PMC3012625; doi:10.1186/1471-2229-10-250)
Supplement: Additional File 5 — Alignment of McDGAT2 and DGAT2 polypeptides from other plant species. [file 1471-2229-10-250-S5.PPT]

## Slide 1
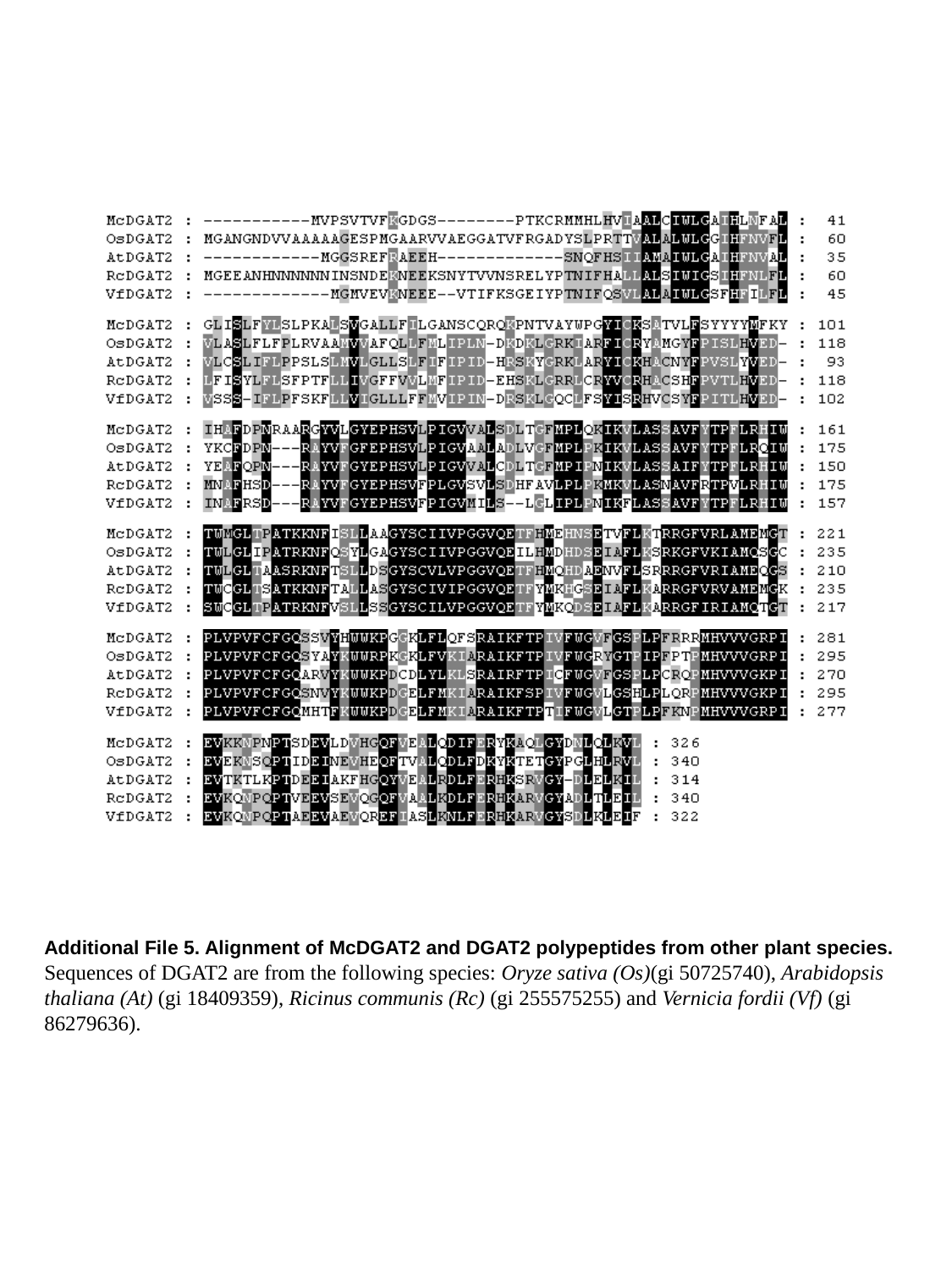

Additional File 5. Alignment of McDGAT2 and DGAT2 polypeptides from other plant species. Sequences of DGAT2 are from the following species: Oryze sativa (Os)(gi 50725740), Arabidopsis thaliana (At) (gi 18409359), Ricinus communis (Rc) (gi 255575255) and Vernicia fordii (Vf) (gi 86279636).
